# Clinician perspectives on OFF periods in Parkinson’s disease: the Spanish PARK-OFF survey-based study

**DOI:** 10.3389/fneur.2026.1869776

**Published:** 2026-09-14

**Authors:** Javier Ruiz-Martínez, Elisabet Mondragón Rezola, Ana Vinagre Aragón, Lara Pardina Vilella, María Sofía Cámara Marcos, David Campo Caballero, On behalf of the PARK-OFF Study group

**Affiliations:** 1 Movement Disorders Unit, Neurology Department, Donostia University Hospital, Donostia-San Sebastián, Spain; 2 Neurodegenerative Diseases Area, ISS Biogipuzkoa, Donostia-San Sebastián, Spain; 3 CIBERNED (Center for Networked Biomedical Research on Neurodegenerative Diseases), Madrid, Spain; 4 Department of Neurosciences, University of the Basque Country (UPV/EHU), Donostia-San Sebastián, Spain

**Keywords:** attitudes, fluctuations, health knowledge, on-demand therapy, Parkinson’s disease, practice, quality of life, surveys and questionnaires

## Abstract

**Purpose:**

The PARK-OFF study aimed to describe participating neurologists’ perceptions of the impact and management of fluctuations in patients with Parkinson’s disease (PD) in Spanish clinical practice.

**Methods:**

PARK-OFF was an ecological and descriptive study based on aggregated data obtained from the experiences of 99 neurologists, collected using a 35-item survey regarding patients with PD and fluctuations seen during the previous month.

**Results:**

On average, 65.9 patients were seen per neurologist in the last month, and neurologists estimated that fluctuations were present in a mean of 46.1% of their patients. The most frequent duration of OFF episodes was 31 to 45 min (41.4%), and neurologists identified wearing-off as the primary contributor to daily OFF time (61.4%). According to the participants, slowness (71.6%), gait impairment (69.8%), and increased falls (67.2%) were considered to have the greatest impact on quality of life during OFF periods. The ON–OFF fluctuations were the least responsive to oral treatments, according to 76.8% of the neurologists. The speed of onset (80.8%) and ease of use (64.6%) were perceived as important factors by the participants in the prescription of an on-demand treatment. Inhaled levodopa was frequently selected as fast (90.9%) and effective (90.9%) on-demand treatment, particularly during wearing-off episodes and morning akinesia, and was associated with high satisfaction among the specialists. Neurologists identified patient education from the specialist (72.5%) as the most useful tool, in their experience, to improve patients’ understanding of the disease and treatment options.

**Conclusion:**

Participating neurologists perceived that fluctuations and OFF periods can impair patients’ daily activities and overall well-being. On-demand treatment with inhaled levodopa was perceived by the neurologists as a useful option in the management of OFF episodes, especially those associated with wearing-off episodes and morning akinesia. Participants considered that initiation of on-demand treatments, along with improved patient education, may support the management of fluctuations in clinical practice.

## 1 Introduction

Parkinson’s disease (PD) is a chronic and progressive neurodegenerative disorder that affects the central nervous system, characterized by the loss of dopaminergic neurons in the *substantia nigra* (1). PD is the second most common neurodegenerative disease worldwide after Alzheimer’s disease, and its prevalence has increased in recent decades, particularly among older adults (2). Motor symptoms in PD include resting tremor, rigidity, bradykinesia, and postural instability, whereas non-motor manifestations include autonomic, sleep, neuropsychiatric, and cognitive symptoms (3). Although PD is progressive, symptom profile and clinical course can vary substantially between individuals. Levodopa remains the standard symptomatic treatment for most patients because of its motor efficacy (4); however, as the disease progresses, many patients develop motor complications such as fluctuations and dyskinesias (5, 6), with approximately 40% of patients who experience this by 4–6 years of treatment (7).

Fluctuations generally refer to alternating periods in which antiparkinsonian medication is providing an adequate clinical effect and function (ON) or reduced benefit (OFF) (8). Fluctuation patterns include wearing-off, ON–OFF transitions, early morning akinesia, nocturnal OFF, partial ON, delayed ON, and dose failures. Non-motor symptoms may precede the onset of motor symptoms in PD, whereas non-motor fluctuations tend to occur later and may worsen during OFF periods, contributing to impaired health-related quality of life (HRQoL) (9). Also, OFF periods may be predictable or unpredictable, with variable onset and duration, which complicates their clinical management.

Currently, several on-demand therapies are available for the treatment of OFF episodes in PD, including subcutaneous apomorphine, sublingual apomorphine, and inhaled levodopa (10, 11). These options differ in route of administration, onset profile, duration of effect, tolerability, and practical considerations, and they may be useful in different clinical scenarios. Recent update from the International Parkinson and Movement Disorder Society highlights the range of available pharmacological approaches for motor fluctuations and the need to adapt treatment strategies according to specific clinical and social patient characteristics (12).

In this scenario, because they avoid some limitations associated with conventional gastrointestinal absorption of oral levodopa, non-oral on-demand therapies may provide a clinically relevant alternative for selected patients (10, 11, 13, 14). Evidence from the SPAN-PD trial has supported the efficacy of inhaled levodopa as an on-demand option for the management of OFF periods, with improvements in motor function observed shortly after administration under controlled conditions (15).

Despite the frequent occurrence of fluctuations and their impact on patients’ daily routines, evidence specifically linking different fluctuation patterns to HRQoL domains remains limited. Previous studies from Europe, France, and the United States have shown that OFF periods and motor fluctuations are associated with worse HRQoL and greater burden in patients with PD (16–18). However, data describing how clinicians perceive and manage these problems in routine national practice remain scarce.

Given the differences in epidemiology and clinical practice across countries, real-world data from national healthcare settings can provide useful contextual insights. In this context, the PARK-OFF survey-based study aimed to characterize neurologists’ perceptions of the burden and management of PD in patients with motor and/or non-motor fluctuations treated in neurology units within the Spanish healthcare system.

## 2 Methods

### 2.1 Study population and design

The PARK-OFF study was an observational, ecological, descriptive survey conducted in real-world clinical practice in Spain. The unit of observation was the participating neurologist. Respondents retrospectively provided aggregate estimates based on their clinical experience during the previous month; no individual patient records, prospectively collected patient-level data, or patient-reported outcome measures were reviewed or analyzed. A total of 100 neurologists from hospitals across multiple regions of Spain were invited to participate. Participating neurologists were informed of study sponsorship and received financial compensation for the survey completion. Eligible participants were neurologists from Movement Disorders Units (MDU) or PD experts, each with a minimum of 5 years of experience and/or an average of at least 10 patients with PD seen per week. Familiarity with the use of inhaled levodopa was a requirement for participation so that respondents could answer treatment-specific items.

### 2.2 Survey and data collection

Between January and March 2025, 99 neurologists (Supplementary Table) completed an online survey. The questionnaire was elaborated as a study-specific set of questions, and no formal psychometric validation was performed. Each neurologist reported data on their clinical experience with patients with PD seen in their consultation during the previous month. The survey, available in the Supplementary material, included a total of 35 questions divided into four sections (Supplementary PARK-OFF Survey): characteristics of participating neurologists (13 questions), aggregated sociodemographic and clinical profile of patients with PD and fluctuations (10 questions), management of PD and its fluctuations (9 questions), and neurologists’ satisfaction with on-demand treatments (3 questions). Relevant items included categorical questions, percentage estimates, and rating scales. No formal patient-level denominator dataset was generated from medical records.

### 2.3 Statistical analysis

A descriptive analysis of study variables was performed. Categorical variables were presented as frequencies and percentages, while continuous variables were presented using mean and standard deviation (SD), as appropriate. Sample size was determined based on precision criteria for the primary descriptive endpoint. Assuming a conservative standard deviation of 25 for a 0–100 scale, a minimum of 100 participating neurologists was estimated to provide a 95% confidence interval with a precision of ±4.9 points. The number of participants answering each question was 99 unless otherwise stated; the surveys that were not fully completed were not considered for analysis. No inferential hypothesis testing was planned or performed. Statistical analyses were conducted using IBM SPSS Statistics, version 29.0 (IBM Corp., Armonk, NY, USA).

## 3 Results

### 3.1 Profile of the participants

Neurologists had a mean (SD) age of 44.7 (10.0) years, and most participants (59.6%) had more than 10 years of experience treating patients with PD (Table 1). Most participants carried out their professional activity in public hospitals (75.8%), with a small proportion working exclusively in private hospitals (1.0%), and 23.2% working in both public and private centers. Neurologists from movement disorders units accounted for 50.5% of the participants, while 49.5% were specialized in PD units. On average, 62.6% of participants required between 30 and 60 min to evaluate a new case of PD, whereas the remaining 37.4% required less than 30 min. When asked about the presence of support, 54.5% of neurologists reported having nursing support. The participant neurologists indicated a mean (SD) number of patients seen in the last month of 65.9 (50.2) patients. From those, a mean of 46.1% (19.2) of the patients presented fluctuations (Table 1), according to the participant neurologists.

**Table 1:** Profile of the neurologists participating in the PARK-OFF study.

Charactristic	Value
Age, mean (SD), years	44.7 (10.0)
Sex, *n* (%), female	53 (53.5)
Years of experience, *n* (%)	
Less than 5 years	14 (14.1)
5–10 years	26 (26.3)
11–19 years	30 (30.3)
20 or more years	29 (29.3)
Patients with PD seen in the last month, mean (SD)	65.9 (50.2)
Patients with fluctuations, mean % (SD)	46.1 (19.2)
With motor fluctuations, mean % (SD)	67.9 (27.7)
With non-motor fluctuations, mean % (SD)	35.4 (26.0)
With motor and non-motor fluctuations, mean % (SD)	42.1 (24.5)

SD, standard deviation.

### 3.2 Clinical and sociodemographic characteristics of the patients

Patient characteristics as described by the neurologists are detailed in Table 2. Neurologists estimated that a mean of 50.8% (22.2) of patients with fluctuations developed them more than 6 years after PD diagnosis, followed by 37.8% (17.6) between 3 and 6 years and 11.5% (14.6) within 2 years or less of diagnosis. Neurologists estimated that oral levodopa was used by a mean of 96.2% (7.1) of patients with PD and fluctuations seen in their practice (Figure 1). Among patients receiving oral levodopa, neurologists estimated that a mean of 51.5% (22.3) developed fluctuations more than 5 years after treatment initiation, 37.8% (17.8) between 2 and 5 years, and 10.7% (12.3) within 2 years or less. The estimated use of inhaled levodopa showed a mean (SD) of 2 (0.8) doses per day, according to the participating neurologists (*N* = 98).

**Table 2:** Characteristics of the patients with PD and fluctuations, according to the neurologist participants (N = 99).

Charactristic	Value
Age, mean % (SD)	
75 or more years	27.7 (14.9)
66–75 years	35.4 (11.6)
56–65 years	24.2 (11.7)
40–55 years	10.4 (8.5)
Less than 40 years	2.3 (4.0)
Sex, mean % (SD), female	45 (9.2)
Family history of PD, mean % (SD)	13.5 (11.7)
Number of OFF episodes per day, *n* (%)	
Less than 1	1 (1.0)
1	8 (8.1)
2	55 (55.6)
3	32 (32.3)
More than 4	1 (1.0)
Unknown	2 (2.0)
Duration of OFF episodes, *n* (%)	
Less than 15 min	3 (3.0)
16–30 min	21 (21.2)
31–45 min	41 (41.4)
46–60 min	29 (29.3)
61 min-2 h	4 (4.0)
More than 2 h	1 (1.0)

SD, standard deviation.

**Figure 1:**
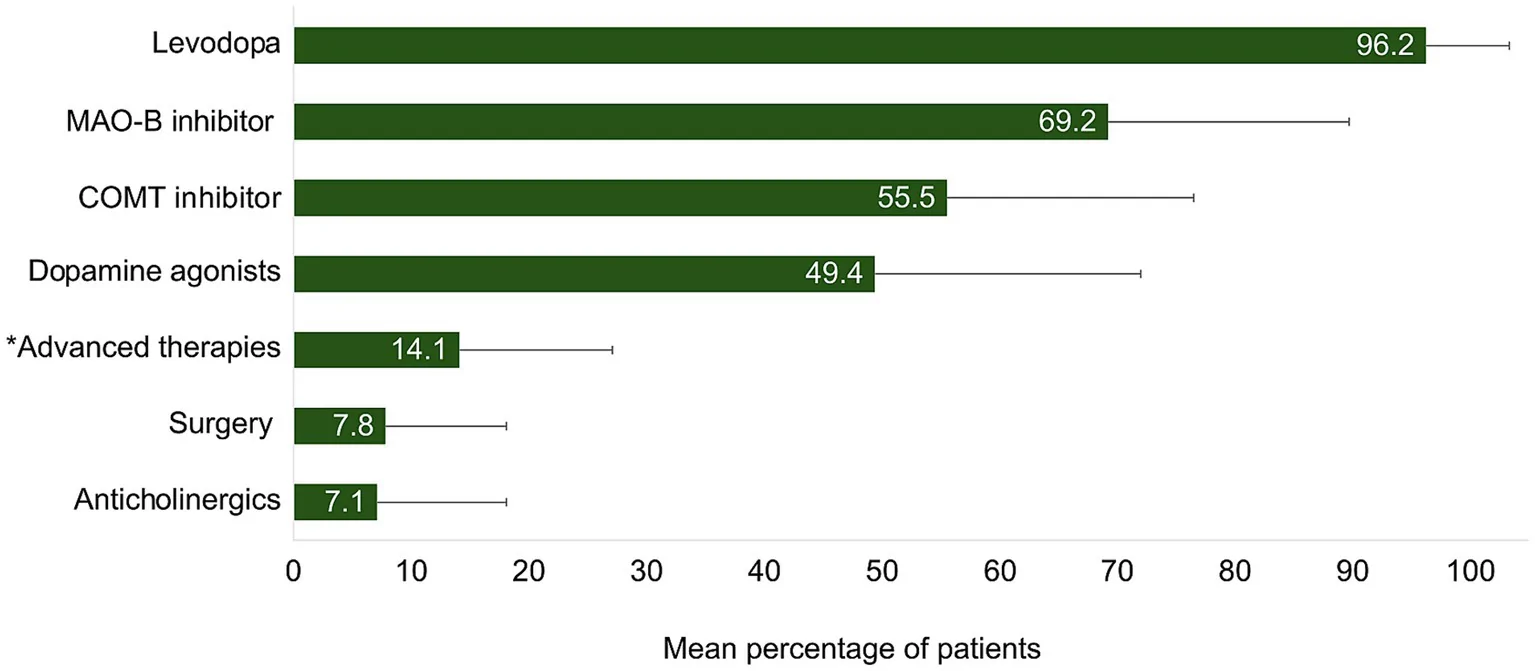
Patients with PD and fluctuations receiving each treatment in the last month, according to the neurologist participants (*N* = 99). MAO-B, monoamine oxidase B; COMT, catechol-O-methyltransferase. Advanced therapies include subcutaneous apomorphine infusion, subcutaneous or intestinal levodopa infusion. Data presented as mean (SD) percentage.

In their experience, neurologists identified independence and employment as the aspects of daily living most affected during OFF episodes on a study-specific 5-point Likert scale (Figure 2). Neurologists considered slowness of movement, gait impairment, and increased falls to be the symptoms associated with the greatest impact on quality of life during OFF periods on a 0 to 100 study-specific scale (Figure 3A). All participants reported having patients with slowness of movement, 98% reported tremor as a symptom present in their patients, and 98% described rigidity during OFF episodes on a 5-point Likert scale (Figure 3B).

**Figure 2:**
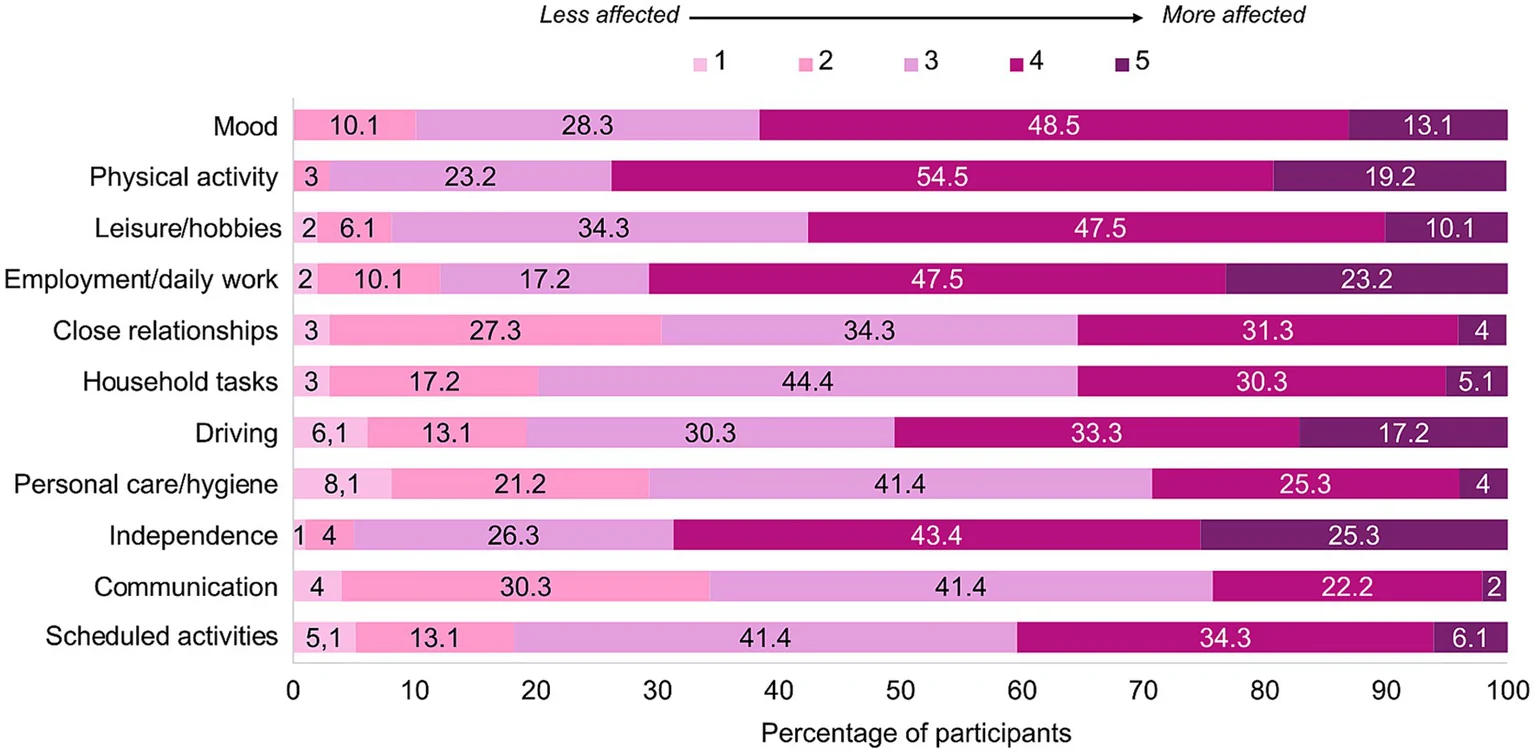
Aspects of life impaired in patients with PD and fluctuations, according to the neurologist participants (*N* = 99). Data are presented as the percentage of responses for each category of a 5-point Likert scale, where 1 indicates less affected and 5 indicates greater affection.

**Figure 3:**
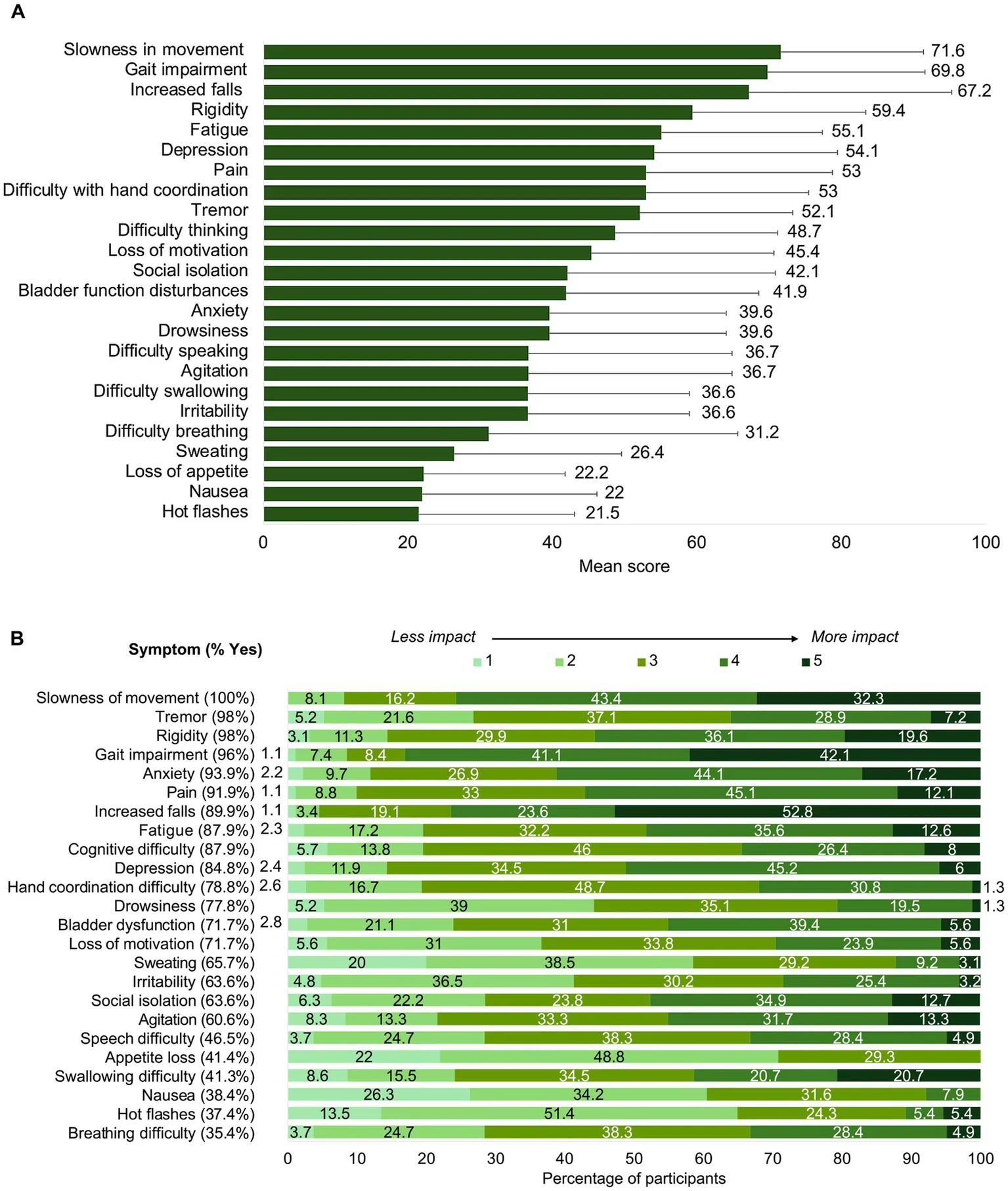
**(A)** Impact on quality of life of symptoms experienced during OFF periods for patients with PD and fluctuations, according to the neurologist participants (*N* = 99). Data presented as mean (SD) score on a 0–100 scale, where 0 indicates no impact and 100 the greatest impact. **(B)** Percentage of neurologist participants reporting patients with any of the symptoms during OFF periods (% Yes) and distribution of impact on quality of life, according to the participants (*N* = 99). Data are presented as the percentage of responses for each category of a 5-point Likert scale, where 1 indicates less impact and 5 indicates greater impact.

### 3.3 Type and management of patient’s fluctuations

The greatest contribution to daily OFF time, as estimated by neurologists, was attributed to wearing-off (mean [SD], 61.4% [22.7]) (Figure 4A). In contrast, ON–OFF fluctuations were identified by 76.8% of neurologists as the least responsive fluctuation pattern to adjustments in oral treatment (Figure 4B). For most participants (76.8%), unpredictable OFF represented less than 25% of OFF periods, whereas 21.2% of participants estimated it accounted for 25 to 50% of OFF periods; only one neurologist (1.0%) reported values above 50%. Neurologists most frequently selected speed of onset (80.8%) and ease of use (64.6%) among the factors relevant for the prescription of an on-demand treatment (Table 3).

**Figure 4:**
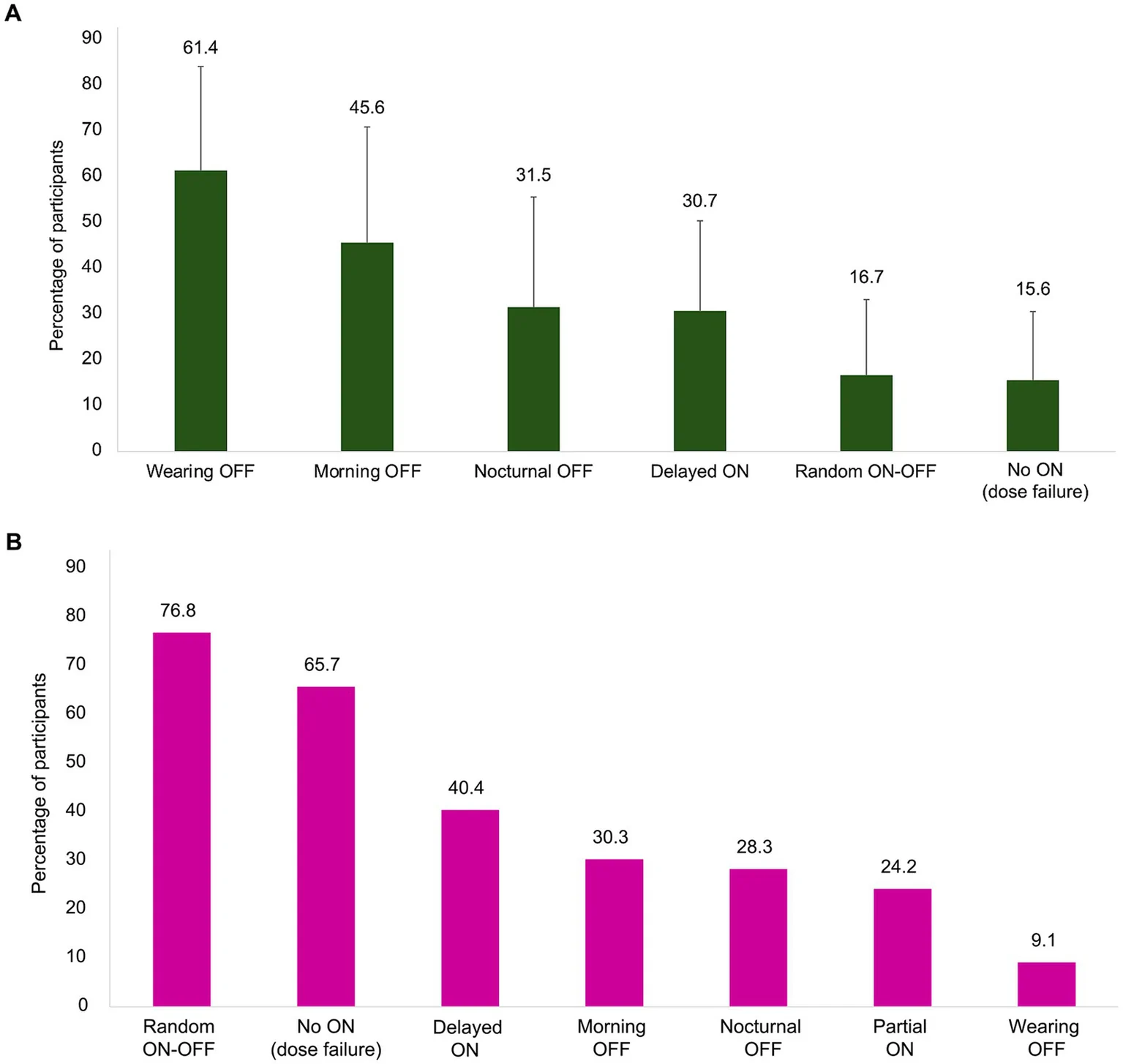
**(A)** Contribution to total daily OFF time of different types of fluctuations, according to the neurologist participants (*N* = 99). Data presented as mean (SD) percentage. **(B)** Less responsive fluctuations to oral treatments, according to the neurologist participants (*N* = 99). Data presented as percentage.

**Table 3:** Neurologists’ perception on prescription of an on-demand treatment and improvement of patient information.

Charactristic	Value
Relevance* in the prescription of an on-demand treatment, *n* (%)	
Speed of onset	80 (80.8)
Ease of use	64 (64.6)
Tolerability	57 (57.6)
Severity of episodes	29 (29.3)
Active compound	23 (23.2)
Preferred route of administration	22 (22.2)
Duration of effect	18 (18.2)
Utility to improve patients’ access to information related to the pathology and treatments, mean % (SD)	
Education from the specialist	72.5 (23.4)
Telephone assistance from nursing staff	49.9 (32.4)
Patient workshops	48.3 (31.9)
Educational physical materials	42.3 (31.0)
Educational videos	39.2 (30.7)

SD, standard deviation. *This item required participants to select 3 factors from a predefined list. Therefore, the reported percentages reflect relative selection frequencies within a constrained choice.

Regarding the management of PD, neurologists rated education from the specialist as the most useful tool to improve patients’ access to information about disease and treatment options, with a mean (SD) utility rating of 72.5% (23.4) (Table 3). Participants unanimously agreed that better information on fluctuation detection, together with education on correct inhaled levodopa use, could improve fluctuation management. Among the perceived benefits of better patient education, 90.9 and 82.8% of participants indicated improved treatment adherence and enhanced functionality, respectively; other potential benefits included better tolerability (50.5%), transition to advanced options (42.4%), and reduction in oral medication (18.2%).

### 3.4 Satisfaction with on-demand inhaled levodopa

From the predefined attributes presented in the questionnaire, participating neurologists selected “fast” (90.9%), “effective for motor symptoms” (90.9%), “good tolerability” (71.7%), “effective for some non-motor symptoms” (62.6%), “predictable” (51.5%), and “easy for patients to administer” (37.4%) in relation to inhaled levodopa. Neurologists most frequently selected wearing-off episodes (86.9%) and morning akinesia (85.9%) as the fluctuation types in which inhaled levodopa may be useful (Figure 5). One participating neurologist reported being dissatisfied, 8 reported a neutral opinion, and 90 (90.9%) reported being satisfied or very satisfied with their experience of inhaled levodopa in routine practice.

**Figure 5:**
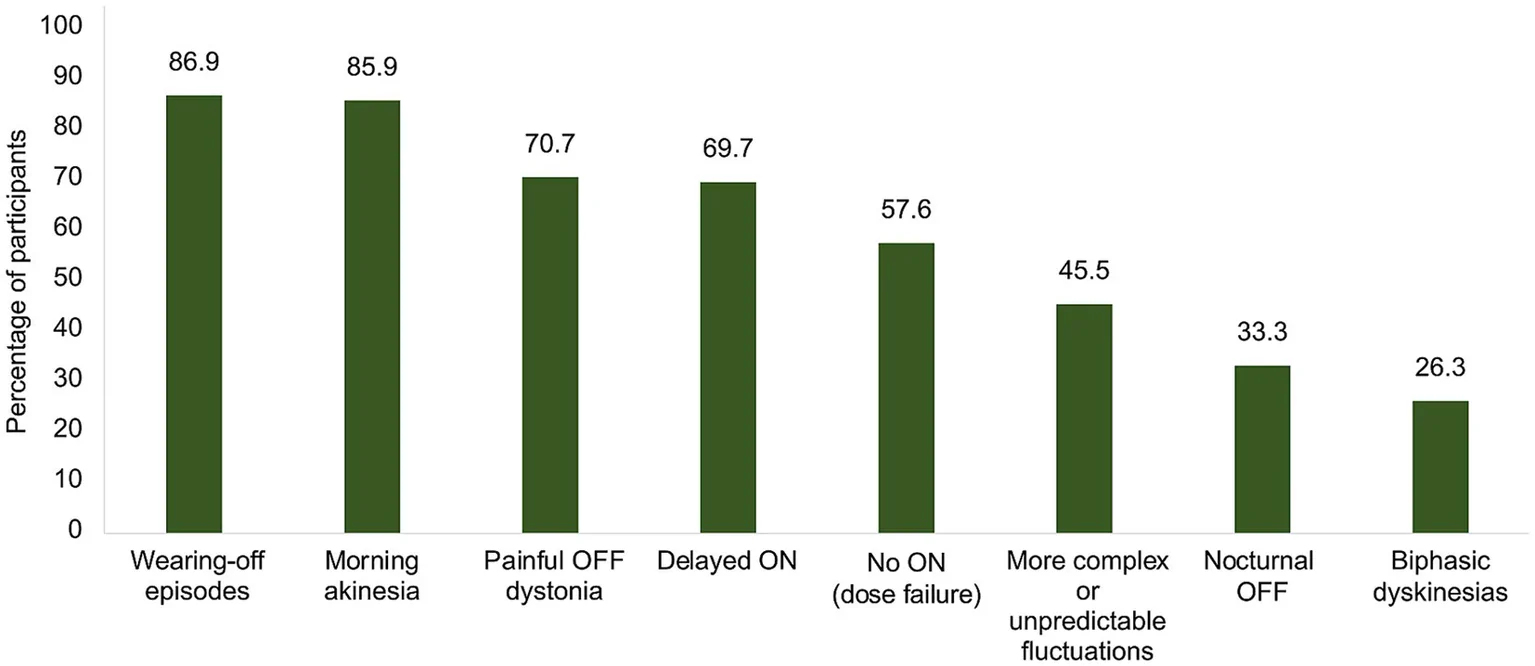
Perception of the effectiveness of on-demand treatment with inhaled levodopa in different types of fluctuations, according to the neurologist participants (*N* = 99). Data presented as percentage.

## 4 Discussion

The PARK-OFF study provides survey-based information on neurologists’ perceptions of the impact and management of motor and non-motor fluctuations in Spanish clinical practice. Its findings should be interpreted in light of the ecological design, as the unit of observation is the neurologist’s aggregate estimate rather than individual patient-level measurement. Accordingly, the findings describe this selected clinician sample and should not be generalized to patients or interpreted as direct estimates of patient burden. Previous literature has shown that motor fluctuations and OFF periods are common complications of PD and are associated with impaired HRQoL and higher burden in affected patients (16–18), but the present survey addresses how neurologists perceive these issues in routine care, adding to the understanding of the complexity in PD. This is particularly relevant as, in the DEEP study, wearing-off was identified more frequently using patient-completed questionnaires than by routine clinical evaluation, highlighting potential discrepancies between clinician perception and patient experience (19). Previous studies have also identified communication gaps between patients and clinicians regarding the recognition and management of OFF periods (20).

In the PARK-OFF study, 99 participating neurologists reported attending a mean of 65.9 patients with PD in the last month. According to their aggregate estimates, neurologists perceived fluctuations as common among patients seen in their practice. They gave the highest perceived-impact ratings to slowness of movement, gait impairment, and increased falls and reported perceived effects across independence, employment, physical activity, and mood. These ratings were obtained using study-specific scales and were not direct patient-reported assessments. These observations are consistent with previous literature showing that OFF periods and motor fluctuations are associated with worse clinical and socioeconomic impact in PD (21). However, in the present study, these findings are based on clinician perceptions rather than direct measurements of HRQoL or economic outcomes.

Survey-based approaches have been previously used to explore the experience and impact of OFF periods from both clinician and patient perspectives (22). Along this line, the PARK-OFF survey highlights the need for improved recognition of non-motor fluctuations, which are clinically relevant yet remain difficult to characterize in routine practice (23). Indeed, although non-motor fluctuations typically emerge after the onset of motor fluctuations (9), a Japanese study evaluated the relationship between motor and non-motor fluctuations and HRQoL, identifying several cases where non-motor fluctuations occurred in isolation (24). Similarly, a Mexican cross-sectional study found that patients without wearing-off or only motor symptoms experienced better well-being, suggesting that non-motor fluctuations might have a higher impact on overall well-being than motor symptoms alone (25). There is also a report indicating that females are at higher risk of suffering non-motor fluctuations, which could present a gender-related pattern (26). In light of this evidence, structured tools, such as patient-reported wearing-off questionnaires (27), have been developed to facilitate the recognition of these fluctuations in clinical practice. Taken together, the literature supports the need for individualized recognition and management of both motor and non-motor fluctuation patterns.

The main category reported by neurologists for OFF frequency was two episodes per day, and the most frequently selected duration category was 31–45 min, followed by 46–60 min, interpreted as aggregated clinician estimates based on recent practice. Nonetheless, the pattern is broadly consistent with previous reports showing that OFF frequency and duration vary across patients and that greater daily OFF burden is associated with poorer HRQoL (18, 28–30). Neurologists identified wearing-off as the main contributor to daily OFF time, whereas unpredictable OFF accounted for less than 25% of OFF periods in most responses and ON–OFF fluctuations were considered least responsive to oral treatment adjustments.

Given that oral levodopa was the most commonly reported treatment among patients seen by the participating neurologists, it is clinically understandable that on-demand non-oral options were considered relevant in selected OFF scenarios. Inhaled levodopa was generally viewed favorably by this sample of neurologists, particularly for wearing-off episodes and morning akinesia. However, these findings should be interpreted cautiously, as the survey included only neurologists familiar with this therapy, which may have favored more positive perceptions. Importantly, on-demand therapies, including inhaled levodopa, subcutaneous apomorphine, and sublingual apomorphine, differ not only in pharmacological characteristics but also in variability of response, onset profile, administration requirements, tolerability, and practical considerations, which may influence their real-world effectiveness and treatment persistence (10, 11). In addition, factors such as patient preference, ease of use, device handling, and treatment burden may play a relevant role in therapeutic decision-making (31). Consequently, treatment selection should remain individualized according to patient-specific characteristics, preferences, clinical context, and treatment goals.

Our data also suggest that participating neurologists consider patient education an important component of fluctuation management. Because recognition of OFF periods may be difficult for some patients, educational interventions and self-management support may help improve communication and treatment use in routine care (32, 33). These findings are limited to clinicians’ perceptions, and no patient-reported assessment of knowledge, preferences, or educational benefit was performed.

We are aware of the limitations of our study. First, it was an ecological survey based on neurologist-reported aggregate estimates rather than direct patient-level measurements, subject to ecological fallacy. Second, responses relied on recall of recent clinical practice over the previous month and were not derived from medical records or patient-reported outcomes, which may have introduced recall and estimation bias. More clinically relevant symptoms may have been more readily recalled, potentially leading to overestimation of their frequency or impact. Third, the questionnaire elaboration was study-specific, and no formal psychometric validation was performed. Fourth, selection bias may have been introduced, as participants were required to be familiar with inhaled levodopa, and affirmation bias may also have arisen from survey items that were predominantly framed with favorable response options. Finally, because the study did not collect patient perspectives directly, the results should not be interpreted as patient-reported burden, satisfaction, or treatment effectiveness.

Despite these limitations, the study provides a comprehensive description of neurologists’ perspectives on OFF periods in routine clinical practice, based on a broad and geographically diverse sample across Spain. The inclusion of both motor and non-motor aspects, as well as treatment-related considerations, adds to the current understanding of the clinical context in which OFF periods in patients with PD are managed in Spain.

## 5 Conclusion

The PARK-OFF study describes how participating neurologists perceive and manage OFF periods and fluctuations in routine practice in Spain. In this selected sample of neurologists familiar with inhaled levodopa, respondents viewed the treatment favorably as an on-demand option, particularly for wearing-off episodes and morning akinesia, and regarded specialist-led education as potentially useful. These results represent clinician perceptions and practice patterns only and should not be interpreted as patient-reported outcomes, patient satisfaction, or evidence of clinical or comparative effectiveness.

## Data Availability

The datasets presented in this article are not readily available because the data supporting this study’s findings are available from the corresponding author upon reasonable request. Requests to access the datasets should be directed to JR-M, javier.ruizmartinez@osakidetza.eus.

## Group member of the PARK-OFF Study group

The PARK OFF Study group, comprised of the following members, who contributed to the collection of data based on their experience and perceptions regarding the management and treatment of patients with PD: Beatriz Tijero, Jone Bocos Portillo, Raquel Vázquez Picón, Elisabet Mondragón Rezola, Anna Reverter Sánchez, Dolores Vilas Rolán, Gonzalo Olmedo Saura, Gabriel Salazar, Mariateresa Buongiorno, Víctor Puente Périz, Almudena Sánchez-Gómez, Celia Painous, Nuria Caballol Pons, Ana Mas Vidal, Mariona Baraldés Rovira, Clara Marzal Espí, Boris R. Capote Acosta, Claustre Pont Sunyer, Tania Delgado Ballestero, Ángela Monterde Ortega, Marcos Alfredo Mandrá, Sonia Escalante Arroyo, María Iciar Gastón Zubimendi, Silvia López Calvo, Alfredo López López, Sara Sánchez Valiente, Adrián Arés-Luque, Elena Rodríguez Martínez, Óscar Durán Borrella, Débora María Cerdán Santacruz, Guilherme Carvalho Monteiro, Olga Fernández Arconada, Ángel Aneiros Díaz, Carlos Cores Bartolomé, Gustavo Fernández Pajarín, Alejandro Rivero de Aguilar Pensado, Carmen Labandeira, Antonio Koukoulis Fernández, Ana Contreras Chicote, Beatriz Álvarez Mariño, Jaime Herreros Rodríguez, Elena Casas Peña, Teresa Maycas Cepeda, Inés Muro García, Carmen Borrue Fernández, Paula Pérez Torre, Ana Fernández Revuelta, Carmen Ribacoba Días, Pilar Sánchez Alonso, Antonio Méndez Guerrero, Emilio Gómez Cibeira, Manuel Delgado Alvarado, Mª Isabel González Aramburu, María Esther Suárez San Martín, David Górriz Romero, Helena Bondía, Carlos Morata Martínez, Jericó Guarner Rodríguez de Sanabria, Berta Claramonte Clausell, Emilio Martínez Maruri, José María Salom Juan, Juan Salas, Ana Catarina de Aragão Soares Homem, Teresa Pérez Saldaña, Eric Freire Álvarez, Erika María Torres San Narciso, Lidia Hernández Rubio, Silvia Martí Martínez, David Dylan García Meléndez, Juan Pablo Cabello de la Rosa, María Fernández Valiente, Noemí Cámara González, Inés Legarda Ramírez, Ana Espino, Abián Antonio Muñoz García, Juan Diego Guerra Hiraldo, Laura Fernández Pérez, Carolina Hernández Javier, Isidro Alexis Acosta Brito, Orestes Andrés Herrera Lorenzo, María Almudena Aguilera del Moral, Juan José Duarte Martín, Olena Romaskevych Kryvulya, Augusto Alejandro Zuhe Colina Rondón, Aldo Costa Valarezo, Alejandro Torres Moral, Toribio Molina Nieto, Francisco Escamilla Sevilla, Jesús Olivares Romero, Javier Gutiérrez García, José María Aguilera Navarro, Alberto Blanco Ollero, Javier Abril Jaramillo, Francisco Pérez Errazquín, María Elena Calzado Rivas, Raúl Espinosa Rosso, Javier Pelegrina Molina, Verónica González Torres, Vicente Serrano Castro.
