# Supplementary material for: Clinician perspectives on OFF periods in Parkinson’s disease: the Spanish PARK-OFF survey-based study
Source: Front Neurol. 2026 Sep 14;17:1869776. doi: 10.3389/fneur.2026.1869776 (PMC13616695; doi:10.3389/fneur.2026.1869776)
Supplement: Supplementary file 1 [file Table_1.pdf]

Supplementary Material

1 Supplementary Table. PARK-OFF Study group.

Autonomous Community	Participant
Basque Country	Beatriz Tijero
	Jone Bocos Portillo
	Raquel Vázquez Picón
	Elisabet Mondragón Rezola
Catalonia	Anna Reverter Sánchez
	Dolores Vilas Rolán
	Gonzalo Olmedo Saura
	Gabriel Salazar
	Mariateresa Buongiorno
	Víctor Puente Périz
	Almudena Sánchez-Gómez
	Celia Painous
	Nuria Caballol Pons
	Ana Mas Vidal
	Mariona Baraldés Rovira
	Clara Marzal Espí
	Boris R. Capote Acosta
	Claustre Pont Sunyer
	Tania Delgado Ballestero
	Ángela Monterde Ortega
	Marcos Alfredo Mandrá
	Sonia Escalante Arroyo
Navarre	María Iciar Gastón Zubimendi
La Rioja	Silvia López Calvo
Aragon	Alfredo López López
	Sara Sánchez Valiente
Castile and León	Adrián Arés-Luque
	Elena Rodríguez Martínez
	Óscar Durán Borrella
	Débora María Cerdán Santacruz
	Guilherme Carvalho Monteiro
	Olga Fernández Arconada
Galicia	Ángel Aneiros Díaz

	Carlos Cores Bartolomé
	Gustavo Fernández Pajarín
	Alejandro Rivero de Aguilar Pensado
	Carmen Labandeira
	Antonio Koukoulis Fernández
Community of Madrid	Ana Contreras Chicote
	Beatriz Álvarez Mariño
	Jaime Herreros Rodríguez
	Elena Casas Peña
	Teresa Maycas Cepeda
	Inés Muro García
	Carmen Borrue Fernández
	Paula Pérez Torre
	Ana Fernández Revuelta
	Carmen Ribacoba Días
	Pilar Sánchez Alonso
	Antonio Méndez Guerrero
	Emilio Gómez Cibeira
Cantabria	Manuel Delgado Alvarado
	Mª Isabel González Aramburu
Asturias	María Esther Suárez San Martín
Valencian Community	David Górriz Romero
	Helena Bondía
	Carlos Morata Martínez
	Jericó Guarner Rodríguez de Sanabria
	Berta Claramonte Clausell
	Emilio Martínez Maruri
	José María Salom Juan
	Juan Salas
	Ana Catarina de Aragão Soares Homem
	Teresa Pérez Saldaña
	Eric Freire Álvarez
	Erika María Torres San Narciso
	Lidia Hernández Rubio
	Silvia Martí Martínez
Castile-La Mancha	David Dylan García Meléndez
	Juan Pablo Cabello de la Rosa
	María Fernández Valiente
Balearic Islands	Noemí Cámara González
	Inés Legarda Ramírez
	Ana Espino
Canary Islands	Abián Antonio Muñoz García

	Juan Diego Guerra Hiraldo
	Laura Fernández Pérez
	Carolina Hernández Javier
	Isidro Alexis Acosta Brito
Melilla	Orestes Andrés Herrera Lorenzo
Ceuta	María Almudena Aguilera del Moral
Extremadura	Juan José Duarte Martín
	Olena Romaskevych Kryvulya
	Augusto Alejandro Zuhe Colina Rondón
Andalusia	Aldo Costa Valarezo
	Alejandro Torres Moral
	Toribio Molina Nieto
	Francisco Escamilla Sevilla
	Jesús Olivares Romero
	Javier Gutiérrez García
	José María Aguilera Navarro
	Alberto Blanco Ollero
	Javier Abril Jaramillo
	Francisco Pérez Errazquín
	María Elena Calzado Rivas
	Raúl Espinosa Rosso
	Javier Pelegrina Molina
	Verónica González Torres
	Vicente Serrano Castro

2 Supplementary Material; PARK-OFF Survey

1. DETAILS OF THE HEALTHCARE PROFESSIONAL

1. Enter your age: _______ years

2. State your sex:
o Male
o Female

3. Select the type of hospital/center (public or private) where you conduct your professional activity:
o Public hospital
o Private hospital
o Public and private hospital (in both models)

4. Which of the following options describes your specialty best?

- Neurologist of the Movement Disorders Unit
- Monographic PD* neurologist
  *Note: Monographic PD neurologist means at least one day a week dedicated to visiting patients with PD.

5. Select the Autonomous Community (region) to which your work center belongs:
- Andalusia
- Aragón
- Asturias
- Balearic Islands
- Canary Islands
- Cantabria
- Castilla-La Mancha
- Castilla y León
- Catalonia
- Ceuta (Autonomous City)
- Extremadura
- Galicia
- Rioja
- Madrid (Community)
- Melilla (Autonomous City)
- Murcia
- Navarre
- Basque Country
- Valencia (Community)

6. Indicate your years of experience in neurology treating patients with PD:
- Less than 5 years
- 5-10 years
- 11-19 years
- 20 years or more

7. On average, how long do you usually take to evaluate a new patient with PD?
- Less than 30 minutes
- Between 30 and 60 minutes
- More than 60 minutes

8. Indicate whether you have nursing support in your neurology office:
- Yes
- No

9. Indicate the approximate number of PD patients that have visited your office in the last month: _____

10. Considering these patients seen in the last month, indicate the approximate percentage of those diagnosed with PD who presented fluctuations: ____%

11. Of the patients with fluctuations described in the last month, indicate the approximate percentage that presented motor fluctuations: ___ %

12. Of the patients with fluctuations described in the last month, indicate the approximate percentage that presented non-motor fluctuations: ___ %

13. Of the patients with fluctuations described in the last month, indicate the approximate percentage that presented both motor and non-motor fluctuations: ___ %

2. SOCIODEMOGRAPHIC AND CLINICAL PROFILE OF THE PATIENTS WITH PD AND CHARACTERIZATION OF THE DISEASE

To complete the information required in this section, consider the global data of your experience with patients with PD presenting motor and/or non-motor fluctuations seen in your office in the last month. Do not evaluate the patient individually but describe the general tendency that may be representative of your usual clinical practice, based on your experience and the protocol in these patients as a group. Please make sure that the information you provide is as close as possible to what has occurred in your usual clinical practice.

14. Age range of the patients with PD and fluctuations seen in your office in the last month:
- Less than 40 years: ___ %
- 40-55 years: ___ %
- 56-65 years: ___ %
- 66-75 years: ___ %
- 75 years or older: ___ %

Note: the total should sum 100. If any condition is not met, indicate 0%.

15. Sex (birth) of the patients with PD and fluctuations seen in your office in the last month:
- Females: ___ %
- Males: ____ %

Note: the total should sum 100. If any condition is not met, indicate 0%.

16. Considering the patients with PD and fluctuations seen in your office in the last month, indicate the percentage with a family history of Parkinson's disease (if not applicable, indicate 0): ___ %

17. Indicate the impact upon the quality of life of your patients, seen in the last month, of their symptoms during the OFF periods described below, using a scale from 0 (no impact) to 100 (maximum impact):
- Fatigue: ___
- Drowsiness: ___
- Tremor: ___
- Stiffness: ___
- Slow movements: ___
- Gait impairment: ___
- Increase in falls: ___
- Hand coordination problems: ___
- Swallowing problems: ___
- Speech problems: ___
- Breathing problems: ___
- Nausea: ___
- Pain: ___
- Anxiety: ___
- Irritability: ___
- Agitation: ___
- Loss of motivation: ___
- Depression: ___
- Social isolation: ___
- Flushing: ___
- Perspiration: ___
- Loss of appetite: ___
- Altered bladder function: ___
- Difficulty thinking: ___

18. Considering the patients with PD and fluctuations seen in your office in the last month, indicate the average time elapsed from the diagnosis of PD to onset of the fluctuations:
- Time from diagnosis 2 years or less: ___ %
- Time from diagnosis 3-6 years: ___ %
- Time from diagnosis over 6 years: ___ %

Note: the total should sum 100. If any condition is not met, indicate 0%.

19. Considering the patients with PD and fluctuations seen in your office in the last month, indicate the average time elapsed from the start of treatment with levodopa to onset of the fluctuations:
- Time from start of levodopa 2 years or less: ___ %
- Time from start of levodopa 2-5 years: ___ %
- Time from start of levodopa over 5 years: ___ %

Note: the total should sum 100. If any condition is not met, indicate 0%.

20. On average, how many OFF episodes do your patients experience on a typical waking day?
- o Less than one episode a day (1 or more a week)
- o 1 episode a day
- o 2 episodes a day
- o 3 episodes a day
- o 4 episodes a day
- o More than 4 episodes a day
- o Don't know

21. On average, how long do the OFF episodes typically last in these patients?
- o Less than 15 minutes
- o Between 16 and 30 minutes
- o Between 31 and 45 minutes
- o Between 46 and 60 minutes
- o Between 61 minutes and 2 hours
- o More than 2 hours

22. Indicate all the symptoms experienced by the patients seen in the last month during the OFF periods, and for each symptom recorded as "yes", score the impact upon quality of life using a 5-point Likert scale (where 1 = no impact and 5 = severe impact):

**Symptom**	**Present: Yes/No/Not sure**	**(if Yes: Impact upon ADL (1-5)**
a. Fatigue		
b. Drowsiness		
c. Tremor		
d. Stiffness		
e. Slow movements		
f. Gait impairment		
g. Increase in falls		
h. Hand coordination problems		
i. Swallowing problems		
j. Speech problems		

k. Breathing problems		
l. Nausea		
m. Pain		
n. Anxiety		
o. Irritability		
p. Agitation		
q. Loss of motivation		
r. Depression		
s. Social isolation		
t. Flushing		
u. Perspiration		
v. Loss of appetite		
w. Altered bladder function		
x. Difficulty thinking		

23. In your experience, and taking into account the patients with PD and fluctuations seen in the last month in your office, to what degree are each of the following aspects of patient life affected by the OFF periods? Score using a numerical scale where 1 = not affected and 5 = greatly affected (the values can be repeated):

- Mood state: ___
- Physical activity: ___
- Leisure time: ___
- Employment/daily work: ___
- Close relations (family, friends, etc.): ___
- Domestic chores: ___
- Driving: ___
- Personal care/hygiene: ___
- Independence: ___
- Communication: ___
- Programmed activities: ___

3. MANAGEMENT OF THE PATIENT WITH PD AND FLUCTUATIONS

To complete the information required in this section, consider the global data of your experience with patients with PD presenting motor and/or non-motor fluctuations seen in your office in the

last month. Do not evaluate the patient individually but describe the general tendency that may be representative of your usual clinical practice, based on your experience and the protocol in these patients as a group. Please make sure that the information you provide is as close as possible to what has occurred in your usual clinical practice.

24. Indicate the percentage of your patients with PD and fluctuations seen in your office in the last month who receive each treatment:

- Levodopa: ___ %
- Dopamine agonists: ___ %
- Monoamine oxidase-B (MAO-B) inhibitors: ___ %
- Catechol-O-methyltransferase (COMT) inhibitors: ___ %
- Anticholinergic agents: ___ %
- Advanced therapies (subcutaneous infusion of apomorphine or subcutaneous or intestinal infusion of levodopa): ___ %
- Surgery: ___ %

Note: since more than one treatment may be prescribed simultaneously, the percentage should not sum 100. If any condition is not met, indicate 0%.

25. Based on your experience, indicate the mean daily doses of your patients treated with inhaled levodopa (a dose is equivalent to 2 capsules): ___

26. Indicate the percentage contribution to the total daily OFF time of the different types of fluctuations in your patients seen in the last month:

- Dose deterioration (wearing-OFF): ___ %
- Morning OFF: ___%
- Delay in ON: ___ %
- No ON (dose failure): ___ %
- Random ON-OFF: ___ %
- Nocturnal OFF:____%

If any condition is not met, indicate 0%. The total may sum more than 100.

27. Indicate which of the following types of fluctuations respond less to oral treatment adjustment (fragmentation of levodopa and/or addition of adjuvant treatments):

- ☐ Dose deterioration (wearing-OFF)
- ☐ Morning OFF
- ☐ Delay in ON
- ☐ No ON (dose failure)
- ☐ Partial ON
- ☐ Random ON-OFF
- ☐ Nocturnal OFF

28. In your patients with PD and fluctuations seen <u>in the last month</u>, what proportion of their OFF periods occur in an unpredictable manner?
- o 0 %
- o Less than 25%
- o Between 25-50%
- o More than 50%

29. Indicate the 3 aspects which in your opinion are of greatest relevance when prescribing on-demand treatment:
- ☐ Drug substance
- ☐ Administration route preference
- ☐ Severity of the episodes (motor fluctuations)
- ☐ Ease of use
- ☐ Speed of onset
- ☐ Tolerability
- ☐ Duration of effect

30. Regarding patient education in dealing with fluctuations, and in the absence of nursing support, indicate the percentage usefulness of each tool for improving patient access to information on his or her disease and treatments:
- Physical educational material (e.g., informative leaflets): ___ %
- Explanation by the specialist: ___ %
- Educational videos: ___ %
- Patient workshops: ___ %
- Telephone support with nursing staff (e.g.: Patient Support Program): ___ %
- Other: ___ % Specify _________

31. In your opinion, could better patient education regarding the detection of fluctuations and training in the use of inhaled levodopa result in improved management of the fluctuations by the patient?
- o Yes
- o No

32. In your opinion, could better patient education regarding the detection of fluctuations and training in the use of inhaled levodopa result in any of the following outcomes?
- ☐ Improved adherence to therapy
- ☐ Improved functionality
- ☐ Improved tolerability
- ☐ Reduction of oral antiparkinson therapy doses
- ☐ Transition treatment towards advanced therapies

4. SATISFACTION WITH ON-DEMAND TREATMENT IN PATIENTS WITH PD AND FLUCTUATIONS

To complete the information required in this section, consider the global data of your experience with patients with PD presenting motor and/or non-motor fluctuations seen in your office in the last month. Do not evaluate the patient individually but describe the general tendency that may be representative of your usual clinical practice, based on your experience and the protocol in these patients as a group. Please make sure that the information you provide is as close as possible to what has occurred in your usual clinical practice.

33. In your opinion, what are the characteristics afforded by inhaled levodopa?

- ☐ Fast
- ☐ Predictable
- ☐ Easy administration by the patient
- ☐ Good tolerability profile
- ☐ Effective against motor symptoms
- ☐ Effective against some non-motor symptoms

34. In your experience, inhaled levodopa may be effective in:

- ☐ Wearing-off episodes
- ☐ Delay in ON
- ☐ No ON (dose failure)
- ☐ More complex or unpredictable fluctuations
- ☐ Morning akinesia
- ☐ Nocturnal OFF
- ☐ Biphasic dyskinesias
- ☐ Painful OFF dystonia

35. As a specialist, indicate your level of satisfaction with on-demand inhaled levodopa for the management of your patients with PD and fluctuations:

- o Very satisfied
- o Satisfied
- o Neutral
- o Unsatisfied
- o Very unsatisfied
